# Early short-term effects on catecholamine levels and pituitary function in patients with pheochromocytoma or paraganglioma treated with [^177^Lu]Lu-DOTA-TATE therapy

**DOI:** 10.3389/fendo.2023.1275813

**Published:** 2023-10-11

**Authors:** Sriram Gubbi, Mohammad Al-Jundi, Sungyoung Auh, Abhishek Jha, Joy Zou, Inna Shamis, Leah Meuter, Marianne Knue, Baris Turkbey, Liza Lindenberg, Esther Mena, Jorge A. Carrasquillo, Yating Teng, Karel Pacak, Joanna Klubo-Gwiezdzinska, Jaydira Del Rivero, Frank I. Lin

**Affiliations:** ^1^ Metabolic Diseases Branch, National Institute of Diabetes and Digestive and Kidney Diseases, Bethesda, MD, United States; ^2^ Department of Endocrinology, Eunice Kennedy Shriver National Institute of Child and Human Development, Bethesda, MD, United States; ^3^ Molecular Imaging Branch, National Cancer Institute, Bethesda, MD, United States; ^4^ Department of Radiology, Memorial Sloan Kettering Cancer Center, New York, NY, United States; ^5^ Center for Health Professions Education, Uniformed Services University of the Health Sciences, Bethesda, MD, United States; ^6^ Developmental Therapeutics Branch, National Cancer Institute, Bethesda, MD, United States

**Keywords:** DOTATATE, PRRT, [177 Lu], [68 Ga], targeted radiotherapy, pituitary, catecholamines, pheochromocytoma and paraganglioma

## Abstract

**Purpose:**

While there are reports of treatment-related endocrine disruptions and catecholamine surges in pheochromocytoma/paraganglioma (PPGL) patients treated with [^177^Lu]Lu-DOTA-TATE therapy, the spectrum of these abnormalities in the immediate post-treatment period (within 48 hours) has not been previously evaluated and is likely underestimated.

**Methods:**

The study population included patients (≥18 years) enrolled in a phase 2 trial for treatment of somatostatin receptor (SSTR)-2+ inoperable/metastatic pheochromocytoma/paraganglioma with [^177^Lu]Lu-DOTA-TATE (7.4 GBq per cycle for 1 – 4 cycles). Hormonal measurements [adrenocorticotropic hormone (ACTH), cortisol, thyroid stimulating hormone (TSH), free thyroxine (FT4), follicle stimulating hormone (FSH), luteinizing hormone (LH), testosterone, estradiol, growth hormone, prolactin], catecholamines, and metanephrines were obtained on days-1, 2, 3, 30, and 60 per cycle as per trial protocol, and were retrospectively analyzed.

**Results:**

Among the 27 patients (age: 54 ± 12.7 years, 48.1% females) who underwent hormonal evaluation, hypoprolactinemia (14.1%), elevated FSH (13.1%), and elevated LH (12.5%) were the most frequent hormonal abnormalities across all 4 cycles combined. On longitudinal follow-up, significant reductions were noted in i. ACTH without corresponding changes in cortisol, ii. TSH, and FT4, and iii. prolactin at or before day-30 of [^177^Lu]Lu-DOTA-TATE. No significant changes were observed in the gonadotropic axis and GH levels. Levels of all hormones on day-60 were not significantly different from day-1 values, suggesting the transient nature of these changes. However, two patients developed clinical, persistent endocrinopathies (primary hypothyroidism: n=1 male; early menopause: n=1 female). Compared to day-1, a significant % increase in norepinephrine, dopamine, and normetanephrine levels were noted at 24 hours following [^177^Lu]Lu-DOTA-TATE dose and peaked within 48 hours.

**Conclusions:**

[^177^Lu]Lu-DOTA-TATE therapy is associated with alterations in endocrine function likely from radiation exposure to SSTR2+ endocrine tissues. However, these changes may sometimes manifest as clinically significant endocrinopathies. It is therefore important to periodically assess endocrine function during [^177^Lu]Lu-DOTA-TATE therapy, especially among symptomatic patients.

**Clinical trial registration:**

https://clinicaltrials.gov/ct2/show/NCT03206060?term=NCT03206060&draw=2&rank=1, identifier NCT03206060.

## Introduction

[^177^Lu]Lutetium-DOTA-DPhe1, Tyr3-octreotate ([^177^Lu]Lu-DOTA-TATE), is an analog of somatostatin with high affinity to somatostatin receptor type 2 (SSTR2) (1), which is overexpressed in various neuroendocrine tumors (NETs), including pheochromocytomas/paragangliomas. Radiolabeled somatostatin analogs have been used successfully in peptide receptor radionuclide therapy (PRRT) and are recommended treatments in societal guidelines for metastatic NETs (2) and for inoperable pheochromocytomas and paragangliomas (PPGL) (3).

As identified in the phase 3 NETTER-1 study, [^177^Lu]Lu-DOTA-TATE is associated with several adverse effects that result primarily from unintended radiation exposure to non-tumor sites (4), and it is known that several endocrine glands (thyroid, pituitary, adrenals, and gonads) express SSTRs (especially SSTR2) on their surfaces (5, 6). In fact, prior dosimetry analyses have demonstrated delivery of significant radiation doses to various endocrine glands after a 7.4 GBq infusion (7). While the clinical effects of PRRT on the endocrine system have been described previously (8, 9), none have investigated the effects of PRRT on the endocrine system in the immediate (<48 hours) time period post administration. This is an important time frame to investigate because patients can exhibit clinical signs of endocrinopathies such as neurohormonal crisis and catecholamine crisis during this early post-administration period, with the package insert for [^177^Lu]Lu-DOTA-TATE indicating that the risk of neurohormonal crisis is in fact the highest during this period (10). In this study, we present the results from a subset of patients enrolled in a prospective trial where the endocrine function and catecholamines levels of PPGL patients treated with [^177^Lu]Lu-DOTA-TATE were evaluated in the immediate post-administration setting at 24h, 48h, and 30 days after each administration of the agent, with the goal of demonstrating that endocrine disruptions can occur very early, perhaps immediately, after the administration of PRRT.

## Materials and methods

This study was a retrospective analysis of prospectively collected data from the trial. Serial blood samples at baseline then at approximately 24h, 48h, and 30 days after each [^177^Lu]Lu-DOTA-TATE administration were obtained from patients aged ≥18 years who were enrolled in the phase 2 [^177^Lu]Lu-DOTA-TATE trial for metastatic/inoperable PPGL (NCT03206060) at the National Institutes of Health (NIH) from October 2017 to March 2020. Hormonal, catecholamine, and metanephrine testing protocols were predetermined by the clinical trial and were performed on day 1 (pre-[^177^Lu]Lu-DOTA-TATE infusion; d1), day 2 (day 1 post-[^177^Lu]Lu-DOTA-TATE infusion; d2), day 3 (day 2 post-[^177^Lu]Lu-DOTA-TATE infusion; d3), day 30 (day 29 post-[^177^Lu]Lu-DOTA-TATE infusion; d30), and day 60 (day 59 post-[^177^Lu]Lu-DOTA-TATE infusion, which is also the day 1 of the next cycle; d60). Pituitary and target organ hormones collected included ACTH, cortisol, thyroid stimulating hormone (TSH), free thyroxine (FT4), gonadotrophs (FSH, LH), testosterone, estradiol, growth hormone (GH), and prolactin, which were measured using immunoassays. Catecholamines and metanephrines were measured using high-performance liquid chromatography. All assays were performed at our institution, except for uninterpretable metanephrine values due to potential interfering substances, which were measured at the Mayo Clinic laboratories using liquid chromatography/mass spectrometry. To ensure accuracy and reproducibility particularly of serum catecholamine levels, all blood samples were drawn at rest and followed a strict protocol where patients laid recumbent for 30 minutes prior and had samples taken from an indwelling venous catheter.

Eligibility criteria for the trial included histologically proven PPGL and documented SSTR+ tumors on a [^68^Ga]Ga-DOTA-TATE positron emission tomography/computed tomography (PET/CT) scan within 12 weeks of treatment. Patients with baseline persistent endocrine abnormalities such as hypogonadism, hypothyroidism, or adrenal insufficiency were excluded from this analysis. [^177^Lu]Lu-DOTA-TATE was administered intravenously every 8 weeks at 7.4 GBq (200 mCi) up to a total of 4 administrations. Concomitant administration of 2.5% Lys/Arg amino acid solution was used for renal protection. The exclusion criteria included the following: 1. Baseline persistent endocrine abnormalities from a clear underlying cause, 2. Gonadotropins in premenopausal women, 3. Unexplained baseline endocrine abnormalities that persisted throughout the course of treatment, and 4. Catecholamine and metanephrine data from patients harboring ‘non-secretory’ PPGL. Further details regarding the exclusion criteria are described in the **Supplementary Information (Section A)**.

Baseline parameters consisting of age, sex, body mass index (BMI), blood pressure (BP) comprising systolic blood pressure (SBP), diastolic blood pressure (DBP), and heart rate (HR), were measured prior to the first [^177^Lu]Lu-DOTA-TATE dose, and then at varying intervals during each cycle: once daily to 2–4 hours/day depending on patient’s clinical stability. Per trial protocol, blood samples were collected from patients on 1, 4, 8, 12, 16, 20, 24, 28, and 32 (+/- 2) weeks of treatment through an indwelling intravenous cannula in a supine, resting state. The timings of the plasma collection were predetermined by the trial protocol at 24-hour intervals post-[^177^Lu]Lu-DOTA-TATE infusion. However, most of these samples were collected between 05:30 AM and 11:30 AM. We also looked into the clinical records of these patients to identify whether any of the patients had undergone an ACTH stimulation test. The study was approved by the institutional review board at the NIH Clinical Center and all patients provided informed consent.

### Statistical analysis

Continuous data were represented as mean ± standard deviation (SD) for normally distributed parameters and as median and interquartile range for skewed data. The longitudinal graphical data were represented as mean ± standard error of mean (SEM). Categorical data were represented as proportions. Longitudinal analysis was performed using linear mixed-effects model analysis. Catecholamine/metanephrine levels were analyzed using two-way repeated measures ANOVA. All longitudinal data were clustered into 5 groups: ‘Day 1’ (D1), ‘Day 2’ (D2), ‘Day 3’ (D3), ‘Day 30’ (D30), and ‘Day 60’ (D60). For example, ‘D1’ comprised d1 values of all cycles (cycle 1 d1, cycle 2 d1, cycle 3 d1, and cycle 4 d1). The ‘D60’ values comprised the d1 values of the subsequent cycles: for example, cycle 2 d1 was not only d1 of the second cycle under ‘D1’ data but was also d60 of the first cycle in the ‘D60’ data. The hormonal measurements were labelled as either ‘high’ or ‘low’ based on the value being above or below the reference range, respectively for the given hormone. Further details on statistics are provided in the **Supplementary Information (Section B)**. All analyses were two-tailed, and the p-value was set to 0.05. Statistical analyses were performed using GraphPad Prism 8 Version 8·3·1 and SAS Version 9.4.

## Results

### Baseline characteristics

Twenty-seven consecutive patients [age: 54 ± 12.7 years, 13 females, BMI: 24.9 (22.7 – 30.9) Kg/m^2^] were enrolled (**Table 1**). At completion of the longitudinal follow-up, 21 (77.8%), 16 (59.3%), and 13 (48.1%) patients had completed the second, third, and fourth doses of [^177^Lu]Lu-DOTA-TATE, respectively. The average pituitary and target gland hormones levels were within the normal reference range. The median baseline levels of plasma normetanephrine and norepinephrine were 5.25x (588 pg/mL; 18 – 112 pg/mL) and 1.04x (826 pg/mL; 84 – 794 pg/mL) the upper limit of normal, respectively. The median baseline epinephrine and metanephrine levels were normal.

**Table 1:** Baseline characteristics of the study population.

Baseline characteristics (n=27)	Values
1. Age (years)	54 ± 12.7
2. Sex: male (%)/female (%)	14 (51.9%) / 13 (48.1%)
3. Body mass index (Kg/m^2^)	24.9 (22.7 – 30.9)
4. Cycles of treatment received: number of patients (%) ○Cycle-1 ○ Cycle-2 ○ Cycle-3 ○ Cycle-4	27 (100%) 21 (77.8%) 16 (59.3%) 13 (48.1%)
5. Adrenocorticotropic hormone (5 – 46 pg/mL; n=25)	24.8 (17.2 – 49.6)
6. Cortisol (5 – 25 mcg/dL; n=25)	11.1 (7.8 – 16)
7. Thyroid stimulating hormone (0.27 – 4.2 microIU/mL; n=24)	2.2 ± 1.12
8. Free thyroxine (0.9 – 1.7 ng/dL; n=24)	1.15 ± 0.19
9. Follicle stimulating hormone (FSH; male; 1 – 11 U/L; n=9)	6.29 ± 3.4
10. Luteinizing hormone (LH; male; 1 – 8 U/L; n=10)	4.67 ± 1.94
11. FSH (postmenopausal female; 22 – 153 U/L; n=7)	70 (51.4 – 83.6)
12. LH (postmenopausal female; 11 – 40 U/L; n=7)	28.8 (25.1 – 40.2)
13. Testosterone (male; 181-758 ng/dL; n=11)	332.3 ± 120.1
14. Estradiol (premenopausal female; 15 – 350 pg/mL; n=5)	96.1 (33.8 – 214.3)
15. Growth hormone (0 – 3 ng/mL; n=26)	0.16 (0.07 – 0.41)
16. Prolactin (4 – 15.2 ng/mL; n=20)	7.65 (2.25 – 13.25)
17. Epinephrine (0 – 57 pg/mL; n=27)	19 (17 – 23)
18. Norepinephrine (84 – 794 pg/mL; n=27)	826 (221 – 4520)
19. Metanephrine (12 – 61 pg/mL; n=27)	45 (26 – 70)
20. Normetanephrine (18 – 112 pg/mL; n=27)	588 (91 – 2285)
21. Dopamine (0 – 25 pg/mL; n=27)	25 (25 – 174)

^Data are represented as mean ± standard deviation or as median (interquartile range).^

### Hormonal variations in the immediate post-treatment period

**Figure 1:**
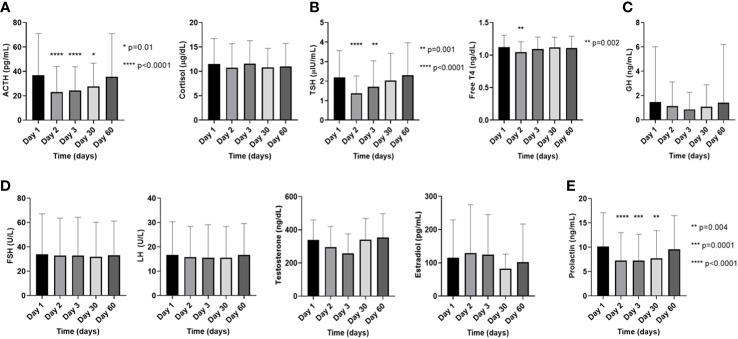
Changes in the pituitary and target endocrine gland hormone levels in the plasma on the day of [177Lu]Lu-DOTA-TATE therapy prior to the infusion (day 1) and during the immediate post-treatment period (day 2 – day 60) in **(A)**. the pituitary-adrenal axis, **(B)**. the pituitary-thyroid axis, **(C)**. the somatotropic axis, **(D)**. the pituitary-gonadal axis, and **(E)**. prolactin. The data on testosterone is exclusively from males and the data on estradiol is exclusively from premenopausal females, while the FSH and LH are combined data from males and postmenopausal females. Each ‘day’ comprises data from all four cycles. Data are represented as mean ± SD.

Regarding prevalence of biochemical abnormalities per patient, estradiol (60%; 3/5 patients – all pre-menopausal women) and prolactin (50%; 10/20 patients) abnormalities were most frequent, followed by testosterone (27.3%; 3/11 patients), ACTH (24%; 6/25 patients), and TSH (20.8%; 6/24 patients) (**Table 2**). Across all measurements, prolactin abnormalities were most frequent (25.4%), with hypoprolactinemia constituting 14.1%, and hyperprolactinemia constituting 11.3%, followed by high FSH (13.1%), high LH (12.5%), and high ACTH (9.8%) (**Table 3**). The descriptions of specific findings from **Table 3** based on individual pituitary-target gland axis is provided in **Supplementary Information (Section C)**. The frequency of all the biochemical endocrine abnormalities on each day of measurement of every cycle is listed in **Supplementary Information (Section D)**. Longitudinal follow-up revealed a reduction in the average levels of several hormones immediately following [^177^Lu]Lu-DOTA-TATE dosing, especially on D2 and D3, with gradual return to pre-[^177^Lu]Lu-DOTA-TATE baseline values at D60 (**Figure 1**). In the pituitary-adrenal axis, compared to D1, the ACTH levels on D2 (D1 = 36.8 ± 34.1 pg/mL vs. D2 = 23.1 ± 21 pg/mL; p<0.0001), D3 (D1 = 36.8 ± 34.1 pg/mL vs. D3 = 24.3 ± 19.4 pg/mL; p<0.0001), and D30 (D1 = 36.8 ± 34.1 pg/mL vs. D30 = 27.7 ± 19.1 pg/mL; p=0.01) were significantly lower, while there were no such corresponding changes in the cortisol levels (**Figure 1A**). In the pituitary-thyroid axis, the TSH on D2 (D1 = 2.2 ± 1.4 microIU/L vs. D2 = 1.4 ± 0.9 microIU/L; p<0.0001), and D3 (D1 = 2.2 ± 1.4 microIU/L vs. D3 = 1.7 ± 1.3 microIU/L; p=0.001) was significantly lower compared to its D1 value, while the FT4 on D2 (D1 = 1.1 ± 0.2 ng/dL vs. D2 = 1 ± 0.2 ng/dL; p=0.002) was significantly lower than the D1 value (**Figure 1B**). There were no significant changes noted on D2, D3, and D30 compared to D1 in the somatotropic axis (GH) (**Figure 1C**), or in the pituitary-gonadal axis (FSH, LH, testosterone, and estradiol) (**Figure 1D**). Compared to D1, significantly lower levels of prolactin were noted on D2 (D1 = 10.2 ± 6.9 ng/mL vs. D2 = 7.2 ± 5.7 ng/mL; p<0.0001), D3 (D1 = 10.2 ± 6.9 ng/mL vs. D3 = 7.3 ± 5.4 ng/mL; p=0.0001), and D30 (D1 = 10.2 ± 6.9 ng/mL vs. D30 = 7.7 ± 5.7 ng/mL; p=0.004) (**Figure 1E**). Five (18.5%) patients had undergone a 250mcg ACTH stimulation test for either a cortisol of <5 mcg/dL or for a cortisol of <10 mcg/dL as clinically indicated, and all the patients demonstrated robust increase in cortisol levels and adrenal insufficiency was ruled out.

**Table 2:** Prevalence of biochemical endocrine abnormalities in the study cohort over four cycles.

Hormone	Number of patients with abnormal results / total number of patients
1. Adrenocorticotropic hormone	6/25 (24%)
2. Cortisol	5/25 (20%)
3. Thyroid stimulating hormone	5/24 (20.8%)
4. Free thyroxine	4/24 (16.7%)
5. Follicle stimulating hormone (males and postmenopausal females)	3/16 (18.8%)
6. Luteinizing hormone (males and postmenopausal females)	4/17 (23.5%)
7. Testosterone (males)	3/11 (27.3%)
8. Estradiol (premenopausal females)	3/5 (60%)
9. Growth hormone	4/26 (15.4%)
10. Prolactin	10/20 (50%)

**Table 3:** Proportion of biochemical endocrine abnormalities among all measurements over four cycles.

Hormone	High	Low
1. Adrenocorticotropic hormone	26/263 (9.8%)	3/263 (1.1%)
2. Cortisol	2/264 (0.8%)	11/264 (4.2%)
3. Thyroid stimulating hormone	17/225 (7.6%)	16/225 (7.1%)
4. Free thyroxine	0/230 (0%)	8/230 (3.5%)
5. Follicle stimulating hormone (males and postmenopausal females)	20/153 (13.1%)	0/153 (0%)
6. Luteinizing hormone (males and postmenopausal females)	21/168 (12.5%)	0/170 (0%)
7. Testosterone (males)	1/122 (0.8%)	10/122 (8.2%)
8. Estradiol (premenopausal females)	3/58 (5.6%)	2/58 (3.4%)
9. Growth hormone	20/274 (7.3%)	0/274 (0%)
10. Prolactin	24/213 (11.3%)	30/213 (14.1%)

**Figure 2:**
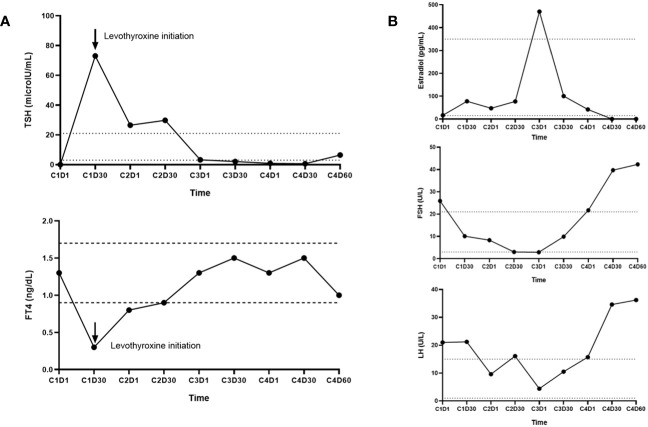
**(A)** Immunoassay measurements of thyroid stimulating hormone (TSH) and free thyroxine (FT4) levels in Patient A who was initially diagnosed with primary thyrotoxicosis prior to [177Lu]Lu-DOTA-TATE therapy initiation and later developed symptomatic primary hypothyroidism 30 days after the first dose of [177Lu]Lu-DOTA-TATE. **(B)** Immunoassay measurements of estradiol, follicle stimulating hormone (FSH), and luteinizing hormone (LH) levels in Patient B who developed secondary amenorrhea and hot flashes close to the 4th cycle dose of [177Lu]Lu-DOTA-TATE. Towards the end of cycle 4, there were elevations in gonadotrophins to menopausal ranges [FSH: 42.3 (menopausal range: 22–153 U/L); LH: 36.2 (menopausal range: 11–40 U/L)], with undetectable estradiol levels (<5 pg/mL; normal menopausal value: <10 pg/mL) suggestive of early menopause (onset of menopause at age <45 years). Estradiol levels demonstrated an initial marked increase (up to 470 pg/mL) followed by a gradual decline to undetectable levels, a phenomenon often observed during perimenopause.

Most biochemical abnormalities were transient and clinically inconsequential. However, clinically symptomatic, and persistent endocrinopathies developed in two patients (**Figure 2**). A 29-year-old male (Patient A) was diagnosed with asymptomatic primary hyperthyroidism due to Hashimoto’s thyroiditis prior to [^177^Lu]Lu-DOTA-TATE therapy (**Figure 2A**). One month after the first dose of [^177^Lu]Lu-DOTA-TATE, the patient developed marked, symptomatic primary hypothyroidism requiring levothyroxine therapy. The pre-treatment [^68^Ga]Ga-DOTA-TATE PET/CT revealed diffuse thyroid uptake with a maximum standardized uptake (SUV_max_) of 14.3, and a diffuse thyroidal uptake of [^177^Lu]Lu-DOTA-TATE was also noted on the 24-hour post-treatment whole body single-photon emission computed tomography (SPECT) scan, with more details described elsewhere (11). A 43-year-old premenopausal woman (Patient B) with normal gonadal function prior to [^177^Lu]Lu-DOTA-TATE therapy developed hot flashes and cessation of menses close to the fourth dose of [^177^Lu]Lu-DOTA-TATE therapy (**Figure 2B**). Towards the end of the fourth cycle, there were elevations in gonadotrophins to menopausal ranges [FSH: 42.3 (menopausal reference range: 22 – 153 U/L); LH: 36.2 (menopausal reference range: 11 – 40 U/L)], with undetectable estradiol levels (<5 pg/mL; normal menopausal value: <10 pg/mL) suggestive of early menopause (onset of menopause at age <45 years) due to hypergonadotropic hypogonadism. The undetectable estradiol was preceded by a marked increase in estradiol levels (up to 470 pg/mL) followed by a gradual decline to undetectable levels, a phenomenon often observed during perimenopause (12).

### Functional imaging characteristics in patients with clinical endocrinopathies

A comparison of the SUV_max_ values of the primary endocrine organs of involvement was performed between patient A and patients who never developed biochemical abnormalities in these respective hormonal axes (‘controls’) on the pre-treatment [^68^Ga]Ga-DOTA-TATE PET/CT scan. Due to poor visualization of ovaries on the functional imaging, patient B was excluded from this analysis. The SUV_max_ of the thyroid in patient A was higher than the mean thyroid SUV_max_ among controls, while the SUV_max_ of the pituitary was lower compared to controls (**Supplementary Information; Section E**). Diffuse thyroid uptake was evident on the pre-treatment diagnostic [^68^Ga]Ga-DOTA-TATE scan and on the post-treatment single-photon emission CT (SPECT) scan, as previously described (11).

### Catecholamine, metanephrine, blood pressure, and heart rate variations

**Figure 3:**
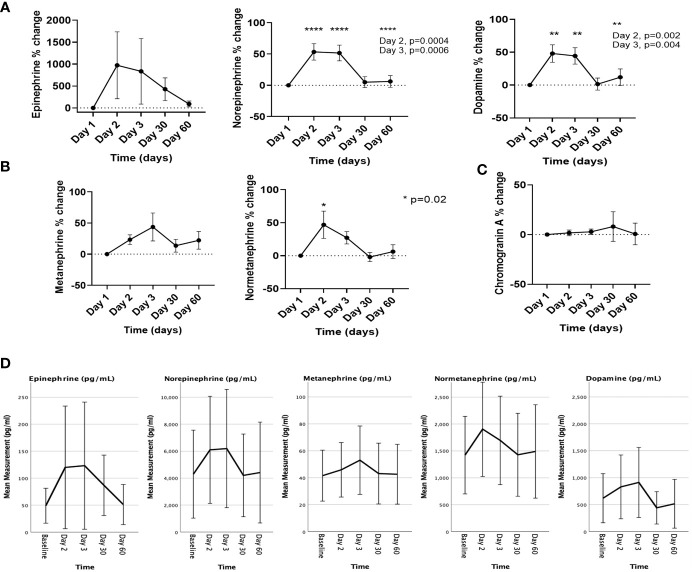
The % changes in the levels of **(A)** catecholamines, **(B)** metanephrines, and **(C)** chromogranin A, and **(D)** the changes in the absolute values of catecholamines and metanephrines on the day of [^177^Lu]Lu-DOTA-TATE therapy prior to the infusion (day 1) and during the immediate post-treatment period (day 2 – day 60). Each ‘day’ comprises data from all four cycles. Data are represented as mean ± SEM.

Compared to D1, significant mean % increase was noted with plasma levels of norepinephrine (53.3% on D2; p=0.0004, and 51.5% on D3; p=0.0006), dopamine (48% on D2; p=0.002, and 44.4% on D3; p=0.004), and normetanephrine (43.9% on D2; p=0.02) (**Figures 3A, B**). The mean % change on D3 for plasma metanephrine tended towards a significant change (41.4%, p=0.06). The % changes for plasma epinephrine and chromogranin A were not significant (**Figures 3A, C**). We also noted an increase in the absolute values in catecholamines and metanephrines, particularly of plasma norepinephrine, normetanephrine, and dopamine at 24- and 48-hours post [^177^Lu]Lu-DOTA-TATE infusion, and the levels returned to baseline by D30 (**Figure 3D**).

**Figure 4:**
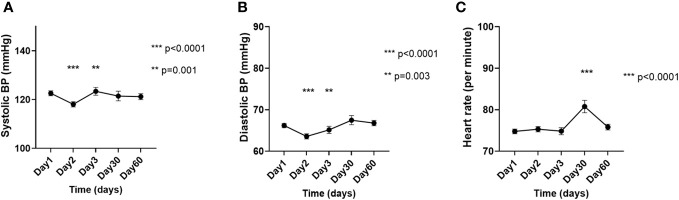
Changes in the **(A)** systolic blood pressure, **(B)** diastolic blood pressure, and **(C)** heart rate on the day of [^177^Lu]Lu-DOTA-TATE therapy, prior to the infusion (day 1) and during the immediate post-treatment period (day 2 – day 60). Each ‘day’ comprises data from all four cycles. Data are represented as mean ± SEM.

Compared to D1, the SBP was significantly lower on D2 (122.6 ± 23.5 mmHg vs. 118.1 ± 20.7 mmHg; p<0.0001), but significantly higher on D3 (122.6 ± 23.5 mmHg vs. 123.4 ± 22.7 mmHg; p=0.001), while the DBP was significantly lower on D2 (66.2 ± 11.6 mmHg vs. 63.6 ± 11.7 mmHg; p<0.0001) and D3 (66.2 ± 11.6 mmHg vs. 65.1 ± 12.3 mmHg; p=0.003), and the HR was significantly higher on D30 (74.8 ± 12.9/min vs. 80.7 ± 15.2/min; p<0.0001) (**Figures 4A-C**).

## Discussion

In this study, we demonstrate that [^177^Lu]Lu-DOTA-TATE treatment is associated with endocrine function fluctuations and catecholamine level surges that are detectable and statistically significant as early as 24 hours and peaking by 48 hours after administration. We also provide prospective evidence that these abnormalities mostly return to pre-treatment levels towards the end of the treatment cycle. These changes are important for the clinician to recognize for safely managing catecholamine surges post [^177^Lu]Lu-DOTA-TATE treatment but also to expect the transient nature of observed pituitary dysfunction.

The longitudinal data in our study revealed a tendency for the average pituitary hormonal levels to decrease on the days immediately following [^177^Lu]Lu-DOTA-TATE infusion, and then return towards baseline by the end of the cycle. These observations suggest that hormonal changes were mostly transient and likely clinically inconsequential. However, clinically evident endocrinopathy developed in 2/27 (7.4%) patients who either had an underlying pre-existing endocrinopathy of target gland (Patient A) or were at risk for developing endocrinopathy (Patient B). SSTRs have been identified in both human thyroid and ovarian tissues (13). Early studies on somatostatin analogs have demonstrated transient suppressive effects on various pituitary hormones a few hours post-administration. Octreotide doses of 50 – 100 mcg have been shown to reduce average GH levels within a few hours in both healthy adults and acromegaly patients (14, 15), as well as reduction in TSH within 6 – 10 hours among patients with TSH-secreting pituitary adenomas (16, 17). A single dose of [^177^Lu]Lu-DOTA-TATE contains about 200mcg (10mcg/mL) of the somatostatin analog component (DOTA-TATE) (18). Although the pharmacokinetic properties of DOTA-TATE have been evaluated (19, 20), it is not clear whether the hormonal secretion-inhibitory properties of the octreotate component of DOTA-TATE are comparable to that of octreotide at similar doses. If this were to be the case, then one could expect similar inhibitory effects on endocrine function, as observed in our study. However, such deductions should be made with caution. Inhibitory effects of octreotide are less obvious on ACTH, cortisol, and FSH secretion (21), yet, we identified ‘low’ values in these hormones, thus suggesting probable mechanisms, independent of the somatostatin analog component of [^177^Lu]Lu-DOTA-TATE, contributing to these effects. The frequently observed pituitary hormonal abnormalities in our study could therefore be due to the effect of [^177^Lu]Lu-DOTA-TATE on the pituitary gland, leading to transient decreases of ACTH, TSH, PRL, while a potential effect of [^177^Lu]Lu-DOTA-TATE on the gonads may have led to a degree of gonadal insufficiency, manifested as increases in FSH and LH, associated with low testosterone in males or to induction of menopause in women (as observed in Patient B) (8, 13).

Hyperprolactinemia/hypoprolactinemia were the most frequently observed abnormalities in our study. Immunohistochemical analysis has demonstrated high-SSTR2 expression in the human lactotrophs, which might explain our observation (22). Similar disruptive effects of [^177^Lu]Lu-DOTA-TATE on other endocrine glands may have contributed towards clinically significant abnormalities in patients-A and B in our study. In a study utilizing [^177^Lu]Lu-DOTA-TATE therapy for gastroenteropancreatic NETs, transient reduction in testosterone and inhibin-B levels, with FSH elevations were identified in men in the first 24 months of therapy, suggesting a potential radiation-induced effect on Sertoli cells (23). Four cycles of 7.4 GBq dose of [^177^Lu]Lu-DOTA-TATE therapy delivers small radiation doses to the adrenals (1.1 Gy), ovaries (0.9 Gy), testes (0.8 Gy), and thyroid (0.8 Gy). These doses are modest, and data from external beam radiation to the pituitary have demonstrated low incidence of hormonal abnormalities with doses <20 Gy, with GH secretion being more susceptible to radiation (<30 Gy), and TSH/ACTH axes being more resistant (up to 50 Gy) (24, 25).

Evidence on [^177^Lu]Lu-DOTA-TATE -associated endocrine disruption has been reported in other studies. Teunissen et al. evaluated endocrine function from baseline up to 24-months follow-up among patients receiving [^177^Lu]Lu-DOTA-TATE (9). At 24 months, the mean total testosterone level in men decreased by 30%, and in postmenopausal women, the mean baseline FSH and LH levels reduced by 16% and 21% respectively from baseline. The mean baseline FT4 levels reduced by 12% at 24 months, with two patients eventually developing primary hypothyroidism. In a phase 2 [^177^Lu]Lu-DOTA-TATE trial on patients with NETs, baseline and yearly pituitary function assessment was performed (8). Plasma IGF-1 decreased by 30% at 48 months, while ACTH increased by 58% during the first year and normalized later. Plasma FSH and LH in males also demonstrated a significant increase within 12 months of [^177^Lu]Lu-DOTA-TATE, followed by normalization of levels, and then followed by an increase in levels after 48 months. In another study, [^177^Lu]Lu-DOTA-TATE therapy in previously normocalcemic patients with NETs (n=47) resulted in significant reductions in serum calcium levels and a significant increase in serum parathyroid hormone (PTH) levels manifesting as secondary hyperparathyroidism when followed up to 6 months after therapy, and 11% of the patients required calcium supplementation (26).

We identified a 2.7-fold higher thyroid uptake on the pre-treatment [^68^Ga]Ga-DOTA-TATE PET/CT scan in Patient A, as compared to the mean uptake among individuals with normal pituitary-thyroid axis in our cohort. Prior studies have shown the thyroid SUV_max_ to be about 4.6 on [^68^Ga]Ga-DOTA-TATE PET/CT scans (27), and in comparison, the uptake (SUV_max_=14.3) in Patient A was 3 times this value. This suggests that there may have been a higher density of SSTRs on the thyroid in Patient A, which may have led to higher uptake of [^177^Lu]Lu-DOTA-TATE, potentially leading to rapid worsening of thyroid function. In fact, thyroid dysfunction (subclinical hypothyroidism) has also been recently reported following the use of α emitter-based PRRT, [^225^Ac]Actinium-DOTA-TATE (28). Our findings warrant careful interpretation as these are findings from a single ‘case’ subject. Dosimetry allows for calculation of thyroid radiation exposure, but dosimetry was not included in our trial’s protocol. A similar mechanism may have contributed to early menopause in Patient B although we could not measure the SUV_max_ in this patient. Further studies evaluating the association between such uptake patterns in other endocrine glands and clinical endocrinopathies are necessary.

We identified significant elevations in catecholamine and metanephrine levels in the initial 30 days following [^177^Lu]Lu-DOTA-TATE dose, findings not previously reported per our knowledge. These elevations started within 24 hours post-administration and had peaked by 48 hours. Direct cytotoxicity from ^177^Lu radionuclide on chromaffin cells of PPGL leading to the spillage of catecholamines and metanephrines could be the most likely mechanism. Moreover, among the patients with secretory PPGL in our cohort, five patients with profoundly high levels of plasma catecholamines were treated with metyrosine, a catecholamine synthesis inhibitor (29), during [^177^Lu]Lu-DOTA-TATE therapy. Even in these patients, catecholamine/metanephrine levels increased following [^177^Lu]Lu-DOTA-TATE infusion, suggesting the increase in plasma catecholamines/metanephrines to be likely due to spillage of preformed molecules from the PPGL. As PPGLs can be SSTR2+ (30), the octreotate component of [^177^Lu]Lu-DOTA-TATE may potentially affect catecholamine production. However, somatostatin analogs decrease catecholamine synthesis from PPGL chromaffin cells (31). Therefore, the somatostatin-mediated effects do not explain the post-treatment increase in catecholamine/metanephrine levels that most likely occurs due to radiation-related tumor disruption. Furthermore, [^177^Lu]Lu-DOTA-TATE-mediated alterations in the dynamics of cytosolic-vesicular catecholamine uptake/reuptake/metabolism, and interactions with adrenal-medullary peptides modulating catecholamine secretion are possible (32), but require further investigation. Another minor contribution to these findings could be from the renoprotective amino acid infusion containing phenylalanine/tyrosine which some of our patients received prior to [^177^Lu]Lu-DOTA-TATE infusion. As phenylalanine/tyrosine are precursors for catecholamine synthesis, they may increase catecholamines in patients with secretory forms of PPGL (33). However, several patients in our study received exclusively lysine/arginine containing amino acid infusions, instead of phenylalanine/tyrosine containing infusions. The BP and HR variability and their correlation with catecholamine/metanephrine changes are challenging to interpret in our study due to patients receiving multiple forms of anti-hypertensive therapy prophylactically or following [^177^Lu]Lu-DOTA-TATE infusion, or due to effects of amino acid infusion on vascular tone. However, hypertension associated with [^177^Lu]Lu-DOTA-TATE have been reported in the literature (34).

Our study has several strengths. Hormonal assays were performed in the same laboratory, thus minimizing inter-institute assay variations. Blood samples were collected through an indwelling intravenous catheter which reduced potential needle trauma-related fluctuations in ACTH and prolactin. Concerns for interference by the non-radiolabeled (‘cold’) somatostatin analogs with [^177^Lu]Lu-DOTA-TATE therapy were unlikely as the trial protocol necessitated the criteria of no initiation/alteration of somatostatin analog therapy within 3 months of enrollment for long-acting octreotide/lanreotide therapies and withholding of short-acting octreotide for 24 hours prior to [^177^Lu]Lu-DOTA-TATE infusion.

However, our study does have limitations. All 4 cycles of [^177^Lu]Lu-DOTA-TATE therapy were completed in 13/27 patients, and data till the end of the 4^th^ cycle was available in 11/27 patients. The list of hormones to be measured were predetermined in the clinical trial protocol, and the data was only available for retrospective analysis. Therefore, effects of [^177^Lu]Lu-DOTA-TATE therapy on several hormones of interest, such as IGF-1, inhibins-A and -B, anti-Müllerian hormone, pancreatic islet hormone function (insulin, hemoglobin A1c), and PTH, could not be assessed. Similarly, per trial protocol, the timings of plasma collection were pre-set at 24-hour intervals post-[^177^Lu]Lu-DOTA-TATE infusion, and the timings of the infusion during the day varied across different cycles and patients. This could have affected the levels of certain hormones measured in our study (such as cortisol and testosterone) which demonstrate diurnal variations in plasma concentration (35, 36). However, most blood samples were obtained no later than 11:30 AM, and we utilized the wide reference range provided by our institutional immunoassays for each hormone such that those hormone levels resulted by our laboratory as ‘high’ or ‘low’ were unequivocal. We also acknowledge the limited utility of random GH measurements due to pulsatile nature of its secretion, as well as the unknown significance of random ACTH measurements in the absence of an underlying/suspected corticotrope or adrenocortical disorder. There is a likelihood for some of the ‘low’ hormonal values to be a result of either a transient inhibition of pituitary hormones by the somatostatin component of [^177^Lu]Lu-DOTA-TATE, or from the dilutional effect from intravenous hydration with 2L normal saline prior to [^177^Lu]Lu-DOTA-TATE infusion. However, this does not explain the several elevated hormonal values that were observed after [^177^Lu]Lu-DOTA-TATE infusion, and therefore, the somatostatin inhibitory effect or the dilutional effect are unlikely to be entirely responsible for the observed changes. Certain ‘low’ values in the thyrotropic and gonadotropic axis hormones could be due to euthyroid sick or eugonadal sick syndromes respectively, which cannot be ruled out in this study population. While all assays were performed at our institution, a few uninterpretable metanephrine values due to potential interfering substances, which were measured at the Mayo Clinic laboratories using liquid chromatography/mass spectrometry. Hormonal evaluation was performed using immunoassays which are highly susceptible to interfering substances and can show wide inter- and intra-assay variations. While the clinically significant endocrinopathies noted in patients A and B persisted till the time of last follow-up in these patients, whether these changes are permanent or whether these were mere coincidental findings remains to be determined.

In conclusion, [^177^Lu]Lu-DOTA-TATE therapy is associated with variations in biochemical endocrine function and with significant changes in plasma catecholamine and metanephrine levels in the immediate post-treatment period. While most hormonal abnormalities are transient and clinically silent, some abnormalities can become profound, clinically significant, and potentially persistent, especially among patients with pre-existing endocrinopathy or those who are at risk for developing an endocrinopathy. Moreover, these observable changes serve as a reminder for potentially more persistent pituitary damage and severe catecholamine surges that may be seen by more powerful agents such as alpha-particle PRRT. Current practice lacks emphasis on evaluating the off-tumor radiation effects in the immediate post-treatment period of [^177^Lu]Lu-DOTA-TATE infusion or other PRRTs. Our data demonstrates that such effects indeed occur post [^177^Lu]Lu-DOTA-TATE infusion. Therefore, early, and serial endocrine function testing should be considered among patients undergoing [^177^Lu]Lu-DOTA-TATE therapy.

## Data availability statement

The original contributions presented in the study are included in the article/**Supplementary Material**. Further inquiries can be directed to the corresponding author.

## Ethics statement

The studies involving humans were approved by National Institutes of Health Clinical Center. The studies were conducted in accordance with the local legislation and institutional requirements. The participants provided their written informed consent to participate in this study.

## Author contributions

SG: Data curation, Formal Analysis, Software, Writing – original draft, Writing – review & editing. MA-J: Formal Analysis, Writing – review & editing. SA: Data curation, Formal Analysis, Software, Writing – review & editing. AJ: Writing – review & editing. JZ: Writing – review & editing. IS: Writing – review & editing. LM: Writing – review & editing. MK: Writing – review & editing. BT: Writing – review & editing. LL: Writing – review & editing. EM: Writing – review & editing. JC: Data curation, Methodology, Supervision, Validation, Writing – review & editing. YT: Formal Analysis, Writing – review & editing. KP: Supervision, Writing – review & editing. JK-G: Supervision, Validation, Writing – review & editing. JD: Supervision, Validation, Writing – review & editing, Methodology. FL: Conceptualization, Funding acquisition, Investigation, Methodology, Resources, Supervision, Validation, Writing – review & editing.
