# Supplementary material for: Early short-term effects on catecholamine levels and pituitary function in patients with pheochromocytoma or paraganglioma treated with [177Lu]Lu-DOTA-TATE therapy
Source: Front Endocrinol (Lausanne). 2023 Oct 11;14:1275813. doi: 10.3389/fendo.2023.1275813 (PMC10598842; doi:10.3389/fendo.2023.1275813)
Supplement: Supplementary file 1 [file DataSheet_1.docx]

Supplementary Material

**Early Short-Term Effects On Catecholamine Levels and Pituitary Function in Patients with Pheochromocytoma or Paraganglioma Treated with [^177^Lu]Lu-DOTA-TATE Therapy**

Sriram Gubbi, MD^1^, Mohammad Al-Jundi, MD^2^, Sungyoung Auh, PhD^1^, Abhishek Jha, MD^2^, Joy Zou, RN^3^, Inna Shamis, CRNP^3^, Leah Meuter, BS^2^, Marianne Knue, CRNP^2^, Baris Turkbey, MD^3^, Liza Lindenberg, MD^3^, Esther Mena, MD^3^, Jorge A Carrasquillo, MD^3,4^, Yating Teng, PhD^6^, Karel Pacak, MD PhD DSc^2^, Joanna Klubo-Gwiezdzinska, MD PhD^1^, Jaydira Del Rivero, MD^5^†, Frank I. Lin, MD^3^†

1. Metabolic Disease Branch, National Institute of Diabetes and Digestive and Kidney Diseases, Bethesda, Maryland, USA
2. Department of Endocrinology, *Eunice Kennedy Shriver* National Institute of Child and Human Development, Bethesda, Maryland, USA
3. Molecular Imaging Branch, National Cancer Institute, Bethesda, Maryland, USA
4. Department of Radiology, Memorial Sloan Kettering Cancer Center, New York, New York, USA
5. Developmental Therapeutics Branch, National Cancer Institute, Bethesda, Maryland, USA
6. Center for Health Professions Education, Uniformed Services University of the Health Sciences, Bethesda, Maryland, USA

†These authors contributed equally to this work and share senior authorship.

**Corresponding author:**

Frank I. Lin, MD

Molecular Imaging Program, National Cancer Institute,

Bldg. 10 Room 13C442, 10 Center Drive,

Bethesda, Maryland, USA 20814

Phone: 240-760-6166

Email: [frank.lin2@nih.gov](mailto:frank.lin2@nih.gov)

ORCID ID: 0000-0003-3539-1454

**Section A. Further details on the exclusion criteria:**

1. Baseline persistent endocrine abnormalities from a clear underlying cause: secondary hypogonadism (n=4; 3 male, one due to high-dose prednisone and opioid treatment and others due to opioid treatment, and 1 female due to opioid treatment), secondary adrenal insufficiency from high-dose prednisone (n=1; male), primary hypothyroidism on levothyroxine (n=2; both female), primary Hashimoto’s thyrotoxicosis (n=1; male), hyperprolactinemia (n=4; all females; prolactinoma in two patients and pituitary hypertrophy in the other two patients).

2. Gonadotropins in premenopausal women as the phase of the menstrual cycle was not known (n=5).

3. Unexplained baseline endocrine abnormalities that persisted throughout the course of treatment: elevated adrenocorticotrophic hormone (ACTH) and cortisol (n=1; male); elevated follicle stimulating hormone (FSH) and luteinizing hormone (LH) (n=1; male); elevated FSH alone (n=1; male); elevated growth hormone (GH) with normal insulin-like growth factor-1 (IGF-1) value (n=1; female), elevated prolactin (n=1; male), and low prolactin (n=2; male).

4. Among patients whose one or more endocrine axes met exclusion criteria, the rest of their endocrine parameters which were normal at baseline were included for the analysis. Also, if any hormonal value was abnormal at baseline (prior to the first [^177^Lu]Lu-DOTA-TATE dose) and remained abnormal for the following few days of testing, but later recovered to normal, the initial abnormal values were excluded as they would not be unambiguously associated with [^177^Lu]Lu-DOTA-TATE therapy.

5. Catecholamine and metanephrine data from patients harboring ‘non-secretory’ PPGL who had normal catecholamine and metanephrine values throughout the [^177^Lu]Lu-DOTA-TATE treatment period, were excluded from longitudinal analysis. In these patients, if one parameter (for example, epinephrine) was not elevated, while the rest of the catecholamines/metanephrines or chromogranin A showed elevated levels, then those were included for further analysis and only the non-elevated parameter (epinephrine) was excluded.

**Section B. Further information regarding the statistical analysis:**

Longitudinal analysis of normally distributed data on hormonal level variations, SBP, DBP, and heart rate was performed using linear mixed-effects model analysis. Longitudinal data on catecholamines, metanephrines, and chromogranin A were represented as % change from the baseline (day 1) of each cycle. In order to evaluate the % change, by using day 1 values as baseline (0% change), the absolute values of catecholamines and metanephrines on a given day ‘n’ were converted into ‘% change’ values [(value _day n_ – value _day 1_ / value _day 1_) x 100]. The distribution of % change data was examined for normality. Linear mixed-effects model analysis was performed to examine whether the mean of % changes from day 1 was significantly different from zero.

For data that were non-parametric even on logarithmic transformation, a box cox transformation was performed, and then a linear mixed-effects model analysis was performed. Post-hoc pair-comparison was performed for longitudinal data using Dunnett-Hsu test. Multivariate analyses were not performed due to the small sample size.

**Section C. Description of the prevalence of endocrine function abnormalities in the study cohort in specific pituitary-target gland axes as listed in Table 3:**

In the pituitary-adrenal axis, 10/11 (90.9%) low cortisol values had corresponding ACTH levels that were normal, and 3 of these low cortisol levels were observed in one patient on d2, d30, and d60 of the 4^th^ cycle without any interfering medications. The remaining 1/11 (0.9%) low cortisol value of 4.6 mcg/dL (normal: 5 – 25 mcg/dL) had a corresponding ACTH value of <5 pg/mL (5 – 46 pg/mL) on cycle 2 d3, and the patient was noted to have not received any interfering medications on or before this day upon medical record review, and both the values returned to normal range without any intervention on subsequent measurements. In the pituitary-thyroid axis, 15/17 (88.2%) of the high TSH values were associated with normal FT4 values, while 1/17 (5.9%) high TSH value (4.5 microIU/mL; normal: 0.27 – 4.2 microIU/mL) had a corresponding low FT4 (0.8 ng/dL; normal: 0.9 – 1.7 ng/dL) value (1/8; 12.5% of all the observed low FT4 values), and both hormones came back to normal ranges without any intervention on subsequent biochemical evaluation. The remaining 7/8 (87.5%) low FT4 values were associated with normal TSH values. All the observed low TSH values (16/16) had corresponding normal FT4 values. While TSH abnormalities were noted in 5/24 (20.8%) patients, these abnormalities were recurring in 3 patients across the 4 cycles, while they were transient in the remaining patients. Such associations in the pituitary-gonadal axis were only reviewed in male subjects as gonadotropin fluctuations could not be accurately assessed in female subjects due to lack of menstrual history data. Out of the 10 low testosterone values, one value (180 ng/dL; normal: 181-758 ng/dL) was associated with an FSH of 18.2 U/L (normal: 1 – 11 U/L), and a normal LH value on cycle 4 d3, and both values returned to normal in this patient on subsequent testing. Six out of ten (60%) low testosterone values were noted in another patient in whom both FSH and LH levels had been elevated even before the first cycle of [^177^Lu]Lu-DOTA-TATE therapy and continued to be elevated through the treatment course (therefore excluded from analysis as per exclusion criteria), but the testosterone levels fluctuated between normal and low values. The remainder of the high FSH and LH values noted in the male subjects were associated with normal testosterone levels.

| **Section D: Table: Prevalence of biochemical endocrine abnormalities for each day of every cycle of [^177^Lu]Lu-DOTA-TATE** **therapy** | | | | | | | | | | | | | | | | |
| --- | --- | --- | --- | --- | --- | --- | --- | --- | --- | --- | --- | --- | --- | --- | --- | --- |
| Hormone | C1D2 | | C1D3 | | C1D30 | | C1D60/C2D1 | | C2D2 | | C2D3 | | C2D30 | | C2D60/C3D1 | |
|  | Increased (%) | Decreased (%) | Increased (%) | Decreased (%) | Increased (%) | Decreased (%) | Increased (%) | Decreased (%) | Increased (%) | Decreased (%) | Increased (%) | Decreased (%) | Increased (%) | Decreased (%) | Increased (%) | Decreased (%) |
| 1. ACTH (5 – 46 pg/mL; n=25) | 1/23  (4.3%) | 1/23  (4.3%) | 0/23  (0%) | 0/23  (0%) | 2/21  (9.5%) | 0/21  (0%) | 2/20  (10%) | 0/20  (0%) | 1/20  (5%) | 0/20  (0%) | 1/20  (5%) | 2/20  (10%) | 2/20  (10%) | 0/20  (0%) | 2/15  (13.3%) | 0/15  (0%) |
| 2. Cortisol (5 – 25 mcg/dL; n=25) | 1/24  (4.1%) | 1/24  (4.1%) | 0/23  (0%) | 0/23  (0%) | 0/21  (0%) | 0/21  (0%) | 0/21  (0%) | 0/21  (0%) | 0/20  (0%) | 1/20  (5%) | 0/20  (0%) | 1/20  (5%) | 0/20  (0%) | 2/20  (10%) | 0/15  (0%) | 2/15  (13.3%) |
| 3. TSH (0.27 – 4.2 microIU/mL; n=26) | 0/23  (0%) | 1/23  (4.3%) | 0/21  (0%) | 1/21  (4.8%) | 2/19  (10.5%) | 0/19  (0%) | 2/18  (11.1%) | 0/18  (0%) | 0/17  (0%) | 1/17  (5.9%) | 1/17  (5.9%) | 1/17  (5.9%) | 2/18  (11.1%) | 1/18  (5.5%) | 1/13  (7.8%) | 1/13  (7.8%) |
| 4. FT4 (0.9 – 1.7 ng/dL; n=26) | 0/24  (0%) | 0/24  (0%) | 0/23  (0%) | 3/23  (13%) | 0/19  (0%) | 0/19  (0%) | 0/18  (0%) | 1/18  (5.6%) | 0/18  (0%) | 1/18  (5.6%) | 0/18  (0%) | 1/18  (5.6%) | 0/18  (0%) | 0/18  (0%) | 0/13  (0%) | 1/13  (7.7%) |
| 5. FSH (male; 1 – 11 U/L; n=12)  (postmenopausal female; 22 – 153 U/L; n=16) | 0/15  (0%) | 0/15  (0%) | 0/15  (0%) | 0/15  (0%) | 0/12  (0%) | 0/12  (0%) | 0/12  (0%) | 0/12  (0%) | 0/12  (0%) | 0/12  (0%) | 0/12  (0%) | 0/12  (0%) | 2/12  (16.7%) | 0/12  (0%) | 1/8  12.5%) | 0/8  (0%) |
| 6. LH (male; 1 – 8 U/L; n=12) (postmenopausal female; 11 – 40 U/L; n=17) | 1/16  (6.3%) | 0/16  (0%) | 0/16  (0%) | 0/16  (0%) | 1/13  (7.7%) | 0/13  (0%) | 1/12  (8.3%) | 0/12  (0%) | 1/13  (7.7%) | 0/13  (0%) | 0/13  (0%) | 0/13  (0%) | 1/13  (7.7%) | 0/13  (0%) | 2/9  (22.2%) | 0/9  (0%) |
| 7. Testosterone (male; 181-758 ng/dL; n=11) | 0/11  (0%) | 0/11  (0%) | 0/11  (0%) | 2/11  (18.1%) | 0/10  (0%) | 0/10  (0%) | 0/8  (0%) | 0/8  (0%) | 0/8  (0%) | 0/8  (0%) | 0/8  (0%) | 1/8  (12.5%) | 0/8  (0%) | 0/8  (0%) | 0/7  (0%) | 1/7  (14.3%) |
| 8. Estradiol (premenopausal female; 15 – 350 pg/mL; n=5) | 1/5  (20%) | 0/5  (0%) | 1/5  (20%) | 0/5  (0%) | 0/4  (0%) | 0/4  (0%) | 0/4  (0%) | 0/4  (0%) | 0/4  (0%) | 0/4  (0%) | 0/4  (0%) | 0/4  (0%) | 0/4  (0%) | 0/4  (0%) | 0/4  (0%) | 0/4  (0%) |
| 9. GH (0 – 3 ng/mL; n=26) | 2/25  (8%) | 0/25  (0%) | 1/25  (4%) | 0/25  (0%) | 1/21  (4.8%) | 0/21  (0%) | 1/21  (4.8%) | 0/21  (0%) | 2/21  (9.5%) | 0/21  (0%) | 1/21  (4.8%) | 0/21  (0%) | 1/21  (4.8%) | 0/21  (0%) | 1/16  (6.3%) | 0/16  (0%) |
| 10. Prolactin (4 – 15.2 ng/mL; n=20) | 2/16  (12.5%) | 1/16  (6.3%) | 2/15  (13.3%) | 2/15  (13.3%) | 3/17  (17.6%) | 0/17  (0%) | 4/17  (23.5%) | 0/17  (0%) | 0/17  (0%) | 1/17  (5.9%) | 1/17  (5.9%) | 2/17  (11.8%) | 2/17  (11.8%) | 2/17  (11.8%) | 3/12  (25%) | 1/12  (8.3%) |

| **Section D: Table: Prevalence of biochemical endocrine abnormalities for each day of every cycle of [^177^Lu]Lu-DOTA-TATE** **therapy (continued)** | | | | | | | | | | | | | | | | |
| --- | --- | --- | --- | --- | --- | --- | --- | --- | --- | --- | --- | --- | --- | --- | --- | --- |
| Hormone | C3D2 | | C3D3 | | C3D30 | | C3D60/C4D1 | | C4D2 | | C4D3 | | C4D30 | | C4D60 | |
|  | Increased (%) | Decreased (%) | Increased (%) | Decreased (%) | Increased (%) | Decreased (%) | Increased (%) | Decreased (%) | Increased (%) | Decreased (%) | Increased (%) | Decreased (%) | Increased (%) | Decreased (%) | Increased (%) | Decreased (%) |
| 1. ACTH (5 – 46 pg/mL; n=26) | 2/14  (14%) | 0/14  (0%) | 1/13  (7.7%) | 0/13  (0%) | 3/13  (23%) | 0/13  (0%) | 1/13  (7.7%) | 0/13  (0%) | 2/13  (15.4%) | 0/13  (0%) | 2/13  (15.4%) | 0/13  (0%) | 2/11  (18.1%) | 0/11  (0%) | 2/11  (18.1%) | 0/11  (0%) |
| 2. Cortisol (5 – 25 mcg/dL; n=26) | 0/14  (0%) | 0/14  (0%) | 1/13  (7.7%) | 1/13  (7.7%) | 0/13  (0%) | 0/13  (0%) | 0/13  (0%) | 0/13  (0%) | 0/13  (0%) | 1/13  (7.7%) | 0/13  (0%) | 0/13  (0%) | 0/11  (0%) | 1/11  (9.1%) | 0/11  (0%) | 1/11  (9%) |
| 3. TSH (0.27 – 4.2 microIU/mL; n=26) | 0/12  (0%) | 2/12  (16.7%) | 2/11  (18.1%) | 1/11  (9.1%) | 1/10  (10%) | 2/10  (20%) | 1/10  (10%) | 1/10  (10%) | 0/10  (0%) | 1/10  (10%) | 1/10  (10%) | 1/10  (10%) | 2/8  (25%) | 1/8  (12.5%) | 2/8  (25%) | 1/8  (12.5%) |
| 4. FT4 (0.9 – 1.7 ng/dL; n=26) | 0/12  (0%) | 0/12  (0%) | 0/11  (0%) | 1/11  (9.1%) | 0/10  (0%) | 0/10  (0%) | 0/10  (0%) | 0/10  (0%) | 0/10  (0%) | 0/10  (0%) | 0/10  (0%) | 0/10  (0%) | 0/8  (0%) | 0/8  (0%) | 0/8  (0%) | 0/8  (0%) |
| 5. FSH (male; 1 – 11 U/L; n=12)  (postmenopausal female; 22 – 153 U/L; n=7) | 1/7  (14.3%) | 0/7  (0%) | 2/7  (28.6%) | 0/7  (0%) | 2/7  (28.6%) | 0/7  (0%) | 3/7  (42.9%) | 0/7  (0%) | 2/7  (28.6%) | 0/7  (0%) | 2/7  (28.6%) | 0/7  (0%) | 3/6  (50%) | 0/6  (0%) | 2/7  (28.6^) | 0/7  (0%) |
| 6. LH (male; 1 – 8 U/L; n=12) (postmenopausal female; 11 – 40 U/L; n=7) | 2/8  (25%) | 0/8  (0%) | 2/8  (25%) | 0/8  (0%) | 2/8  (25%) | 0/8  (0%) | 1/8  (12.5%) | 0/8  (0%) | 1/8  (12.5%) | 0/8  (0%) | 2/8  (25%) | 0/8  (0%) | 2/7  (28.6%) | 0/7  (0%) | 2/8  (25%) | 0/8  (0%) |
| 7. Testosterone (male; 181-758 ng/dL; n=12) | 0/7  (0%) | 2/7  (28.6%) | 0/7  (0%) | 1/7  (14.3%) | 0/7  (0%) | 1/7  (14.3%) | 0/6  (0%) | 0/6  (0%) | 0/6  (0%) | 0/6  (0%) | 0/6  (0%) | 2/6  (33.3%) | 0/6  (0%) | 0/6  (0%) | 1/6  (16.7%) | 0/6  (0%) |
| 8. Estradiol (premenopausal female; 15 – 350 pg/mL; n=5) | 1/4  (25%) | 0/4  (0%) | 0/3  (0%) | 0/3  (0%) | 0/3  (0%) | 0/3  (0%) | 0/3  (0%) | 0/3  (0%) | 0/3  (0%) | 0/3  (0%) | 0/3  (0%) | 0/3  (0%) | 0/3  (0%) | 1/3  (33.3%) | 0/2  (0%) | 1/2  (50%) |
| 9. GH (0 – 3 ng/mL; n=27) | 0/15  (0%) | 0/15  (0%) | 1/14  (7.1%) | 0/14  (0%) | 3/13  (23.1%) | 0/13  (0%) | 1/13  (7.7%) | 0/13  (0%) | 3/13  (23.1%) | 0/13  (0%) | 1/13  (7.7%) | 0/13  (0%) | 1/11  (9.1%) | 0/11  (0%) | 0/11  (0%) | 0/11  (0%) |
| 10. Prolactin (4 – 15.2 ng/mL; n=26) | 2/11  (18.1%) | 3/11  (27.3%) | 3/11  (27.3%) | 3/11  (27.3%) | 1/10  (10%) | 3/10  (33.3%) | 1/11  (9.1%) | 1/11  (9.1%) | 0/11  (0%) | 3/11  (27.3%) | 0/11  (0%) | 3/11  (27.3%) | 0/10  (0%) | 2/10  (20%) | 0/10  (0%) | 3/10  (30%) |

**Section E:** Comparison of SUV_max_ of thyroid and pituitary glands between patient-A and five controls with no known abnormalities in the thyrotropic axis. Data are represented as mean ± standard error of mean.

| **Subject** | **Thyroid SUV_max_** | **Pituitary SUV_max_** |
| --- | --- | --- |
| 1. Patient A | 14.3 | 23.4 |
| 2. Controls (n = 5) | 5.3 ± 0.9 | 26 ± 6.6 |
